# Studying non-alcoholic fatty liver disease with zebrafish: a confluence of optics, genetics, and physiology

**DOI:** 10.1007/s00018-012-1037-y

**Published:** 2012-06-08

**Authors:** Amnon Schlegel

**Affiliations:** 1 University of Utah Molecular Medicine (U2M2) Program, University of Utah School of Medicine, 15 North 2030 East, Building 533, Room 3240B, Salt Lake City, UT 84124 USA; 2 Department of Internal Medicine, Division of Endocrinology, Metabolism and Diabetes, University of Utah School of Medicine, Salt Lake City, UT USA; 3 Department of Biochemistry, University of Utah School of Medicine, Salt Lake City, UT USA

**Keywords:** Zebrafish, Lipid metabolism, Hepatic steatosis, Non-alcoholic fatty liver disease

## Abstract

Obesity is a public health crisis. New methods for amelioration of its consequences are required because it is very unlikely that the social and economic factors driving it will be reversed. The pathological accumulation of neutral lipids in the liver (hepatic steatosis) is an obesity-related problem whose molecular underpinnings are unknown and whose effective treatment is lacking. Here I review how zebrafish, a powerful model organism long-used for studying vertebrate developmental programs, is being harnessed to uncover new factors that contribute to normal liver lipid handling. Attention is given to dietary models and individual mutants. I speculate on the possible roles of non-hepatocyte residents of the liver, the adipose tissue, and gut microbiome on the development of hepatic steatosis. The highlighted work and future directions may lead to fresh insights into the pathogenesis and treatment of excess liver lipid states.

## Introduction

### Pandemic obesity

The post-World War II rise in obesity continues unabated [1], and it is clear that unanticipated and previously unrecognized players in regulating energy homeostasis must be discovered in order to combat the illnesses that follow from calorie excess. Indeed, the current pharmacologic armamentarium used in treating obesity’s attendant illnesses (e.g., type 2 diabetes mellitus, hypertension, dyslipidemia, atherosclerotic vascular disease) has seen some of its gains negated by increased adiposity [2]. Thus, there is pressing need for systematic and comprehensive strategies for identifying novel genes that participate in all aspects of energy homeostasis.

Special attention is needed for phenotypes of excessive and ectopic lipid accumulation because of the poorly understood reasons driving their development, and the toxic effects of ectopic lipid accumulation on the whole organism. More concretely, multiple lines of evidence indicate adipose tissue has a finite capacity to safely store neutral lipids. The spill-over of lipid metabolites from over-stuffed adipocytes into the circulation leads to a host of problems, including insulin resistance, hepatic steatosis, intramyocellular liposis, accelerated atherosclerosis, hypertension, and β-cell dysfunction [3]. While metabolic syndrome and type 2 diabetes mellitus are the most prominent manifestations of deranged lipid storage, it has become widely recognized that obesity compounds or causes several other conditions including high-output heart failure, restrictive lung disease, certain cancers, degenerative joint disease, and chronic kidney disease [4].

### Non-alcoholic fatty liver disease

Of the obesity-related disease processes just mentioned, it is important to underscore that excessive liver accumulation of lipids (hepatic steatosis) is present in a large fraction of obese persons [5]. Alarmingly, hepatic steatosis is found in 30 % of the general population, is present in nearly two-thirds of patients with diabetes mellitus, and is seen in over 90 % of very obese persons seeking weight-reduction surgery [6]. Comprehensive reviews cataloging what has been learned from human genome-wide association studies on hepatic steatosis [7, 8], and from monoallelic human disorders marked by hepatic steatosis [8] leave us with a frustrating picture of the genetic underpinnings of the inappropriate accumulation of liver fat. First, the frequency of Mendelian inheritance of hepatic steatosis is very low, and affected persons often have striking, additional phenotypes not found in the general population. Similarly, in large, prospectively gathered cohorts of otherwise seemingly healthy adults only one, repeatedly reproduced, robust association with a coding polymorphism (I148M) in a lipid-modifying enzyme gene *PNPLA3* has been identified [9, 10]. Targeted deletion of the *Pnpla3* gene in mice does not cause hepatic steatosis [11, 12], suggesting that the human polymorphism does not cause a loss of function. In support of a dominant or gain-of-function effect of the PNPLA3^I148M^ mutation, overexpression of the mutated human PNPLA3 (but not wild-type PNPLA3) causes increased mouse liver lipid accumulation [13]. This gain-of-function observation reconciles an early report that PNPLA3^I148M^ has only modestly decreased triacylglycerol hydrolase activity [9] (initially attributed to prevention of triacylglycerol break-down) with more recent work showing that PNPLA3^I148M^ has increased lysophosphatidic acid acetyltransferase activity relative to wild-type PNPLA3 (and is thereby a major enzyme of triacylglycerol synthesis that has only modest triacylglycerol hydrolase activity) [14].

Other associations between human polymorphisms and hepatic steatosis have smaller attributable risk or are not reproducible in more diverse cohorts [15, 16], leaving us with a frustratingly incomplete picture of which genes might contribute to this complex phenotype. This frustration mirrors the clinical heterogeneity of the disorder being studied and the lack of effective treatments for it. Hepatic steatosis is the first step in a spectrum of disorders that encompasses inflammation (steatohepatitis), fibrosis (cirrhosis), and cancer (hepatocellular carcinoma) [17]. These inter-related conditions are collectively referred to as non-alcoholic fatty liver disease (NAFLD). There are limited therapeutic options for permanently ameliorating hepatic steatosis [18, 19] and there are no methods for reversing hepatic fibrosis, or preventing hepatocellular carcinoma due to NAFLD [20]. Indeed, the highest-quality clinical trial shows very modest benefits for taking pioglitazone, an insulin-sensitizing Peroxisome proliferator activated receptor gamma agonist with multiple, long-term safety concerns [19]. Paralleling this disappointing result are the genome-wide association observations that while many persons with NAFLD are obese and even have diabetes, genes directly involved in insulin signaling have not been implicated in the pathogenesis of this condition [7, 8]. Thus, very high direct and indirect health costs are sustained by inappropriate accumulation of neutral lipids in the liver and little by way of pharmacologic agents is available to reverse this.

Ultimately, the inability to treat NAFLD reflects a lack of detailed knowledge of what triggers it [7], and what drives its progression. The first step of NAFLD is the inappropriate accumulation of triacylglycerol in the hepatocyte [21]. This accumulation may be due to excessive de novo hepatic lipid production, decreased hepatic secretion of very low density lipoprotein particles, diminished β-oxidation of fatty acids in the liver, more subtle defects in regulating energy homeostasis including insulin resistance or central nervous system nutrient sensing, a combination of these factors, or some yet-to-be-appreciated mechanisms. Since each of these possibilities could be amenable to therapeutic exploitation, understanding their regulation is paramount.

Here I review the novel insights into hepatic lipid metabolism gleaned from work with the model organism *Danio rerio*, underscoring mechanistic studies that bring new molecular players into focus, and highlighting new areas that need to be explored.

## Zebrafish: convergence of physiology, optics, and genetics

### Overview

**Fig. 1:**
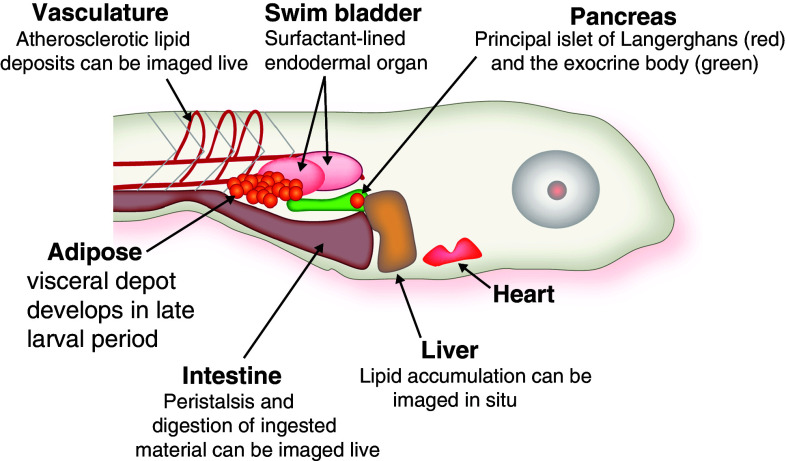
Anatomy of late larval zebrafish. Within the first week of life, zebrafish larvae have functional circulatory and digestive systems. The latter is under neuroendocrine control, as represented by function pancreatic islets of Langerhans, which synthesis insulin and glucagon. After 5–7 days of feeding (beginning 5 dpf), they accumulate lipids in the visceral adipose depot. At this age, the animal remains sufficiently transparent to allow for whole-mount imaging of internal organs

The strengths of zebrafish as a model organism for studying vertebrate development—facilitated by external fertilization, relatively rapid development, optically transparency, transgenic feasibility, and genetic tractability—have been widely recognized for over two decades [22]. This long and successful track record of advances in understanding vertebrate development through the analysis of wild-type and mutant zebrafish has opened new avenues of mechanistic research into multiple disease processes [23]. Because it has the conventional vertebrate body plan that includes central and autonomic nervous systems, digestive organs, and adipose tissue, zebrafish is well suited for studies modeling human energy metabolism, as well [24] (Fig. 1).

### Lipid packaging and transport

The zebrafish utilizes a lipid-packaging machinery that is conserved among metazoans to transport neutral lipids from their sites of absorption (intestine and yolk) or synthesis (liver) to peripheral organs. The molecular players involved include apolipoproteins [25, 26] and apolipoprotein processing enzymes [27, 28]. These pathways have been reviewed previously, and will not be rehearsed again [24]; however, it is worth mentioning that following release into the circulation, lipoproteins are modified in zebrafish blood by a machinery that is also highly conserved in evolution. For instance, zebrafish carry an ortholog of the human Cholesteryl Ester Transfer Protein (*CETP*) gene. A *Cetp* ortholog is frustratingly absent in commonly used rodent models, rendering the study of atherosclerosis difficult in these systems since the animals are inherently resistant to atherosclerosis [29]. The retention of the *cetp* gene in zebrafish causes the circulating lipoproteins to resemble human lipoproteins in abundance and composition, and this contributes to the susceptibility of zebrafish to atherosclerosis when placed on a high-cholesterol diet [30]. Furthermore, the deposition of subintimal cholesterol can be monitored in real time, in live animals [31]. The ability to study atherosclerosis in zebrafish should help in examining the mechanisms underpinning the well-known association of human NAFLD and atherosclerosis [32].

### Dietary studies

In addition to propensity to atherosclerosis when placed on high-cholesterol diets, zebrafish develop obesity, hypertriglyceridemia, hepatic steatosis, and characteristic adipocyte gene expression changes when over-fed their normal diet [33]. These effects can be reversed by caloric restriction. While complex metabolic phenotyping with methods like calorimetry, glucose tolerance and insulin tolerance testing, and stable isotope kinetic analysis is not yet feasible in zebrafish, one potential strength of this organism in generating diet-induced phenotypes is the ability to screen potential therapeutic compounds in a large-scale manner. High-throughput methods for performing chemical screens in zebrafish are now available, and it is conceivable that metabolic phenotypes like amelioration of hepatic steatosis could be studied in such a platform [34]. There is promising preliminary data in support of this hope: diet-induced hepatic steatosis can be ameliorated by high-lycopene and β-carotene-containing tomato extract [35]. The molecular mechanism of this reversal of hepatic steatosis in this model includes induction of genes encoding lipid oxidizing proteins. This study of naturally occurring, complex extracts of vegetables suggests that high-throughput screening of synthetic chemical libraries may achieve similar, if not greater, effects.

Along similar lines, a recent study of Liver X receptor (Nr1h3) (Lxr) agonists finds that these drugs behave similarly in zebrafish as they do in rodent pre-clinical models [36]. Specifically, treatment of zebrafish larvae with Lxr agonists cause hepatic steatosis, a finding that has frustrated drug development exploiting other beneficial properties of Lxr activation [37]. Screening of new Lxr modulators for hepatic steatosis in zebrafish might accelerate drug development.

### Genetic screens

While the dietary and pilot pharmacological studies of hepatic steatosis in zebrafish are encouraging, the identification of mutants with hepatic steatosis holds the potential to identify completely unanticipated pathways contributing to liver lipid metabolism, and possibly to design of rational therapeutic strategies. To date, five mutants with hepatic steatosis have been reported. The mutated genes and phenotypes of the affected animals are summarized in Table 1. One mutant was generated by forward (unbiased) chemical mutagenesis [38]. Two are from a library of lethal, retrotransposable element insertion mutants [39, 40]. The fourth is a spontaneous retrotransposable element insertion mutation found in some commonly used laboratory strains [41]. The final is a “directed,” chemical mutagen-induced lesion in a gene of interest to the investigators who described it [42].

**Table 1:** Summary of zebrafish hepatic steatosis mutants

Mutant	Gene symbol	Protein	Function	Phenotypes	Mechanisms involved in hepatic steatosis
*foie gras* (*fgr*)	*trappc11*	Trafficking protein particle complex 11	Involved in endoplasmic reticulum and Golgi traffic	Hepatic steatosis hepatomegally hepatocyte nuclear degeneration Lethal	Generation of endoplasmic reticulum stress. Induction of lipid biosynthetic enzyme gene expression
*ducttrip* (*dpt*)	*ahcy*	*S*-adenosyl-homocysteine hydrolase	Methyl donor metabolic enzyme	Hepatic steatosis Liver and pancreas degeneration Lethal	Induction of lipid biosynthetic enzyme and inflammatory gene expression
*hi559*	*cdipt*	Cytidine diphosphate-diacylglycerol-inositol 3-phosphatidyltrasnferase	Phospholipid synthesis	Hepatic steatosis Lethal	Endoplasmic reticulum stress Mitochondrial morphological defects
*red moon* (*rmn*)	*slc16a6a*	Solute carrier family 16, member 6a	β-hydroxybutyrate transporter	Fasting hepatic steatosis Viable	Failure to secrete ketone bodies causes carbon atom storage in triacylglycerol
	*skt11*	Serine/threonine kinase 11 (LKB1)	Phosphorylation of the nutritional-sensor AMP-kinase	Fasting hepatic steatosis Glycogen depletion Viable	Inadequate activation of Prka (AMP-activated protein kinase) causing incomplete suppression of de novo lipogenesis and cholesterol biosynthesis

**Fig. 2:**
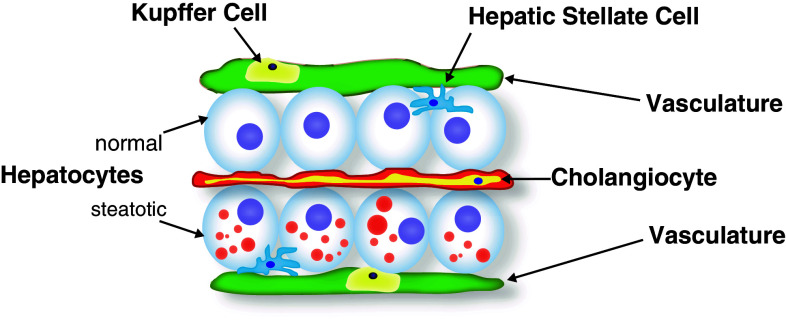
Histology of late larval zebrafish liver. The polarized hepatocytes are sandwiched between the apical bile space in which the cholangiocytes reside, and a basolateral space occupied by scattered hepatic stellate cells and bounded by the vasculature. Within these blood vessels, resident macrophages (Kupffer cells) reside. In hepatic steatosis, the hepatocytes fill with cytoplasmic lipid droplets (*red circles*). Drawing based on models and data presented in Refs. [57, 61]

Below, I summarize the major findings with each mutant and discuss the implications of their identification and characterization. All reported mutants have been characterized during the late larval period, a time in development when the liver already assumes a mature architecture with numerous cell populations present and active (Fig. 2). Two of these mutants have also been examined for adult phenotypes [38, 41].

The first zebrafish hepatic steatosis mutant to be reported is *foie gras* (*fgr*) [39]. This mutant was identified in a re-screening of a library of lethal mutations for liver size: *fgr* mutants display hepatomegaly, marked by hepatocyte nuclear degeneration and steatosis. The retrotransposon insertion into the affected gene causes a very early truncation of the encoded protein. The *fgr* mutant is phenocopied by a translation initiation-disrupting antisense morpholino oligonucleotide: *fgr* is a null mutation. The function of the encoded Fgr protein was revealed in studies using transfected mammalian cells and *Drosophila* mutants. The Fgr protein is a higher metazoan-specific component of the Trafficking Protein Particle Complex (it is subunit 11, or TRAPPC11), a multimeric protein complex involved in endoplasmic reticulum (ER)-to-Golgi apparatus trafficking [43]. Interestingly, in *Drosophila*, the orthologous gene is required for memory formation: the *fgr* ortholog *gryzun* gene was identified in a screen for *Drosophila* mutants with impaired memory formation. In this study, the Gryzun protein was shown, again, to be an important component of the TRAPP complex [44]; notably, however, the human ortholog of TRAPPC11 (formerly called C4orf41) was shown to be required for Golgi exit of a secretory protein and for maintenance of Golgi architecture, but not for ER to Golgi transit of the model cargo protein. Future studies should explore how loss of this TRAPP component in zebrafish gives rise to hepatic steatosis. Additional experiments are required to resolve where and when this complex acts in the secretory machinery, assessing specifically for cell-specific functions to this TRAPPC component. For instance, is very low density lipoprotein particle egress disrupted by mutation of Trappc11? If so, is there also defective intestinal secretion of chylomicrons? More broadly, what is the role of endoplasmic reticulum stress in generating the hepatic steatosis seen in *fgr* mutants, particularly as it relates to activation of master transcription factors regulating lipid biosynthesis [45]. Since the TRAPPC11 gene is broadly expressed, compound phenotypes affecting other organs might preclude full analysis in zebrafish mutants.

Another homozygous mutant reflecting this potential pitfall of zebrafish screens (numerous phenotypes beyond the desired hepatic steatosis) is *ducttrip* (*dtp*). The *dpt* mutant was recovered in a chemical mutagen-induced screen for defects in exocrine pancreatic [46]. This mutant demonstrates normal early pancreatic differentiation, but the ductal network and acinar marker expression were not normal later in development. When the identity of the gene responsible for this phenotype was determined by positional cloning, additional experiments were performed to demonstrate that *dpt* mutants have hepatic steatosis and liver degeneration [38]. The gene affected in this mutant is *ahcy*, which encodes *S*-adenosylhomocysteine hydrolase, an enzyme critical to generation of methyl donors for use in numerous biological processes including nucleic acid, protein, and lipid biochemistry. The *dtp* mutation induces increased expression of genes encoding enzymes of de novo lipogenesis and it activates an inflammatory reaction, marked by increased Tumor necrosis factor-α production. These results confirm nicely the evolutionarily central role of methyl donor biology in normal liver function: numerous studies in rodents employ a methionine and choline-deficient diet to trigger a rapid and severe steatohepatitis. Fortuitously, heterozygous adult *dtp* carriers show a steatosis phenotype, indicating that while the homozygous mutants have an intractable degeneration phenotype, the haploinsufficient adults present an experimentally tractable system for addressing biochemical and immunological questions stemming from altered methyl donor biology.

Another demonstration that zebrafish livers employ highly conserved molecular pathways to maintain homeostasis is found in the *hi559* mutant, which bears an inactivating insertion in the *cdipt* gene [40]. This mutation impairs phosphatidyl inositol (PI) synthesis, and lack of this critical phospholipid (presumably) in the liver triggers hepatocyte ER stress marked by activation of the unfolded protein response and hepatocyte apoptosis by a mechanism awaiting full elucidation. The mitochondrial morphology is altered in *hi559* mutant hepatocytes, but it is not known whether this change affects fatty acid oxidation. Since whole-larval PI levels are not altered prior to death, and the exocrine pancreas appears normal in *hi559* mutants, these findings suggest that de novo PI synthesis in the hepatocyte is critical to maintaining normal ER and mitochondrial form and function. These findings add to an emerging literature on the interface of ER and mitochondrial homeostasis and hepatic lipid metabolism [47], and prompt further work examining signaling pathways requiring this phospholipid for normal hepatic lipid regulation.

My group recently reported the positional cloning and characterization of the *red moon* (*rmn*) mutant [41]. The mutated gene encodes an integral plasma membrane protein Slc16a6a that had not been characterized previously. We found that this protein is a transporter for the major fuel of fasting, β-hydroxybutyrate. As the fasted animal exhausts its hepatic glycogen stores, fatty acids and amino acids partially oxidized into acetoacetate and its reduction product β-hydroxybutyrate. These “ketone bodies” are secreted into the circulation in a 1:3 molar ratio. Unlike longer acyl chain fatty acids, ketone bodies are suitable substrates for use by the brain during periods of fasting. Mutation of this hepatocyte exporter of β-hydroxybutyrate causes diversion of liver-trapped ketogenic precursors into triacylglycerol, as revealed by radiotracer analysis. Underscoring the importance of Slc16a6a to normal fasting physiology, previously fed *rmn* mutants are more sensitive to death by starvation than are wild-type larvae. Our unbiased, forward genetic approach reveals a heretofore unrecognized critical step in fasting energy metabolism: hepatic ketone body transport. Since β-hydroxybutyrate is both a major fuel and a signaling molecule in fasting [48], the discovery of this transporter provides an opportunity to modulate circulating levels of ketone bodies in metabolic diseases.

## Future directions

### Genetic background considerations

In our study, never-fed *rmn* mutants and their wild-type siblings died between 12 and 13 days post-fertilization (dpf). The animals were hybrids deriving from AB × Singapore mutagenized animals that were subsequently crossed to the mapping WIK strains [49]. The spontaneous *rmn* mutation was carried, we determined, by the Singapore strain. This strain is evolutionarily related most closely to the AB strain and the genomic reference Tübingen (Tü) strain [50]. Indeed, the Tü isolate used in the genomic assembly harbors the same retrotransposable element insertion as our Singapore isolate does in the *slc16a6a* gene. Our Tü stock at the University of Utah carries the *rmn* mutation, as well.

The *rmn* mutant’s pedigree raises an underappreciated area of zebrafish investigation, namely the relative lack of concern for genetic background needs to be revisited, particularly as more mutants are generated through high-throughput methods. For instance, mutants derived from the Hubrecht Institute’s targeting induced local lesions in genomes (TILLING) project have a complex genetic composition: the very large number of mutagenized males were of the common Tupfel long fin (TL) strain, which we found does not develop hepatic steatosis if never fed [28]. The TL strain TILLING mutation carriers were outcrossed to the common AB strain, or a hybrid AB × München strain, however [51]. This latter strain is not widely available, and its genetic contribution might account for the fasting steatosis at 11 dpf seen in wild-type siblings of *serine/threonine kinase 11* (*stk11*) [42]. The *stk11* gene encodes Liver kinase B1, a serine/threonine kinase upstream of the evolutionarily central energy sensor AMP-activated protein kinase, whose activation is required during fasting to mobilize glucose and to suppress de novo lipogenesis and cholesterol biosynthesis [52]. The *stk11* mutants show hepatic glycogen depletion and hepatic steatosis 7 dpf, a point in development when the wild-type siblings show adequate glycogen content and no steatosis. This is a gratifying result considering an extensive body of work done in mice with mutations in the orthologous gene [53]. This particular zebrafish mutant might be useful in a chemical screen for modulators of the central AMP-activated protein kinase energy-sensing pathway. Nevertheless, in contrast to our work with the AB × Singapore × WIK wild-type siblings of *rmn* mutants, the TL × AB (×München, possibly) wild-type siblings of *stk11* mutants do develop hepatic steatosis in the never fed state by 11 dpf. Thus, future studies of all zebrafish mutants should keep genetic background considerations in mind, and selected wild-type strains should be meticulously examined prior to the initiation of a project. Fortunately, new genomics data is emerging and this may guide strain selection: recent work comparing the often-mutagenized AB, and Tü strains, a genuinely wild isolate from Bangladesh, and the highly polymorphic (with regard to microsatellite markers) WIK strains reveals that zebrafish have very high levels of copy number variations (CNVs) relative to higher vertebrates, and that the Tü strain has the highest CNVs of all strains examined [54]. These findings make a strong case for using “composite” stocks of animals derived from intercrossing of many individuals of different strains in order to evenly distribute the number and type of CNVs. Such a highly polymorphic, periodically refreshed wild-type stock might minimize the effect of genetic background on phenotypes, but it will require intensive coordination to achieve across all laboratories using zebrafish.

### More screens or more phenotypes?

While forward genetics has identified new and potentially “drugable” pathways to modulate hepatic lipid mass, there are several challenges to working with zebrafish that need to be addressed if this system is to yield maximum fruit. First, screens need to be followed by robust phenotypic characterization. Mechanisms of metabolic derangements will be uncovered only by using all the advantages of the system listed above in tandem with new technologies that allow for detailed characterization. Assays that are standard in rodent models must become standard in zebrafish. Body composition, sophisticated lipidomic approaches, and classical physiological methods must be applied where possible. Introducing bias into screens for viable mutants (i.e., rejecting mutants that die as larvae) may lower the overall yield of mutants recovered, but it may increase the likelihood that such mechanistic biochemical experiments can be performed. It is worth noting that live screening for hepatic steatosis with new lipid reporters may accelerate scoring of the phenotype and allow for more detailed phenotypic characterization of lipid dynamics [55].

### What else is in there?

By the end of the larval period, the zebrafish liver assumes an anatomic configuration—hepatocyte polarization, establishment and function of a biliary network, and vascularization—reflective of mature organ function [56]. Beyond the hepatocytes, cholangiocytes, and endothelial cells required to achieve this architecture, the liver has additional cell types that contribute to lipid homeostasis (Fig. 2). For instance, the innate immune reaction to hepatocyte injury is mediated by the resident macrophage population (Kuppfer cells), and these cells are central to the development of steatohepatitis [57]. Fortunately, robust fluorescent reporters of this cell type are established [58], making live imaging to monitor their presence, number, and (potentially) activation in NAFLD feasible. Similarly, hepatic stellate cells, another cell type activated in response to hepatocyte injury that is critical to the fibrotic response at the far end of the NAFLD spectrum [59], can now be investigated using the strengths of this model system [60]: a powerful transgenic driver for labeling this population of cells is available. These cells, like hepatic stellate cells of higher vertebrates, store retinoids, and proliferate in response to ethanol exposure. Thus, the zebrafish system affords the opportunity to combine optical and genetic properties to study critical steps of NAFLD pathogenesis beyond the inciting event of steatosis.

### Adipose tissue biology

Along similar lines, an integrated, “extra-hepatic” view of the genes identified in mutants with hepatic steatosis must be taken (Fig. 1). For instance, in those viable mutants with hepatic steatosis, the consequences of the molecular lesion on the adipose tissue need to be explored in a manner similar to that already achieved with diet-induced obesity models. Also, because the ontogeny of zebrafish visceral adipose has been described [61, 62], transgenic tools to label these adipocytes for live imaging should be developed. Not only would such reagents allow interrogation of adipocyte developmental biology, but they would provide a window into simultaneous evaluation of multiple lipid-handling organs. Finally, better tools for interrogating insulin signaling (e.g., antibodies, transgenic reporter lines) are also sorely lacking for this organism and need to be prepared.

### Gut microbes

In humans and in mice, the gut microbiome has profound effects on nutrition [63]. An emerging appreciation for the role of the gut microbiome in NAFLD makes it an attractive area of investigation [64]. The core microbiome of the zebrafish intestine has been defined [65]. Thus, this model organism should be used to explore the form and function of the gut microbiome on nutrient sensing and processing using the optical, genetic, and physiological properties highlighted above. Specifically, the interplay of gut microbes with the development or amelioration of hepatic steatosis should be examined in future studies.

## Conclusions

The zebrafish is emerging as a powerful system for discovering and investigating new pathways that contribute to hepatic lipid metabolism and exploring the mechanisms of NAFLD. The identification, cloning, and analysis of several mutants with hepatic steatosis demonstrate that this approach will uncover important biological players. A combination of new genetic, optic, and physiologic tools and approaches should yield still more novel insights.
